# Association Between External Training and Self-Reported Competence Improvement Among County Hospital Healthcare Providers: A Cross-Sectional Study from China

**DOI:** 10.3390/healthcare14152281

**Published:** 2026-07-27

**Authors:** Siyi Hu, Penghao Fan, Siyuan Zhang, Lingling Ye, Tingting Lei, Chao Rong

**Affiliations:** 1 School of Humanities and Management, Zhejiang Chinese Medical University, 260 Baichuan Street, Hangzhou 311400, China; 202521114611002@zcmu.edu.cn (S.H.); 202421114611012@zcmu.edu.cn (S.Z.); 202421114611010@zcmu.edu.cn (L.Y.); 202321114611004@zcmu.edu.cn (T.L.); 2 School of Nursing, Zhejiang Chinese Medical University, 548 Binwen Road, Hangzhou 310053, China; 202321114611003@zcmu.edu.cn; 3 Zhejiang Cultural Research Institute of Chinese Medicine, Zhejiang Chinese Medical University, 548 Binwen Road, Hangzhou 310053, China

**Keywords:** county hospital, healthcare providers, external training, propensity score matching

## Abstract

Background: Training has been shown to be associated with competence improvement among healthcare providers. This study aimed to evaluate the relationship between external training and self-reported competence improvement among county hospital healthcare providers in the context of China’s “Thousand-County Project” policy. Methods: A multi-center cross-sectional survey was conducted from June to September 2025, involving 605 healthcare providers from 30 county hospitals across six provinces in China. Propensity score matching was used to control for observed confounding, and multivariable ordinal logistic regression was applied to estimate the odds ratio (OR) and 95% confidence interval (CI) for the association between post-policy external training and self-reported competence improvement, using 5-point Likert scale as the ordered outcome. Interaction tests and a series of sensitivity analyses were conducted to assess robustness and explore potential effect modification. Results: Matching effectively balanced the distribution of covariates between the two groups. Ordinal logistic regression showed that participants who received post-policy external training reported significantly higher self-reported competence improvement than those who did not (OR = 3.36, 95%CI: 1.74–6.48, *p* < 0.001). Policy awareness was also strongly associated with self-reported competence improvement (OR = 4.50, 95%CI: 2.53–7.99, *p* < 0.001), and a significant interaction indicated that this association was substantially stronger among those who were aware of the policy (*p* for interaction = 0.043). Sensitivity analyses, including cluster-robust standard errors and alternative propensity score specifications, yielded consistent results, supporting the robustness of the findings. Conclusions: Post-policy external training was significantly associated with higher self-reported competence improvement among county hospital healthcare providers, and this association appeared stronger among those with greater policy awareness. To better support such initiatives, policymakers could consider strengthening financial support for external training, while hospitals might optimize staffing arrangements to facilitate training participation. Additionally, improving policy dissemination may help raise healthcare providers’ awareness of relevant policies.

## 1. Introduction

China’s county hospitals serve as the backbone of the primary healthcare system, covering approximately 70% of the country’s population [1]. However, they have long been plagued by inadequate medical resources, talent shortages, and low technical capacity [2], leading patients to seek care at high-level hospitals in large cities, a pattern that increases the financial burden and exacerbates overcrowding in tertiary facilities [3].

To address these challenges, the National Health Commission launched the “Thousand-County Project” in 2021, aiming to bring at least 1000 county hospitals up to tertiary-level service capacity by 2025. A core strategy outlined in the policy is building a high-quality talent workforce, with particular emphasis on increasing external training opportunities for healthcare providers. This strategic choice reflects the recognition that county hospitals often lack internal expertise to deliver high-quality in-house continuing education, making external training at higher-level institutions a more feasible mechanism for knowledge transfer and capacity upgrading.

In contrast, most studies have focused on in-hospital interventions, such as internal lectures, simulation training, and online courses [4,5,6,7], which are fundamentally different in nature from external training. External training is defined as educational activities undertaken by healthcare providers at higher-level medical institutions outside their own workplace. Drawing on experiential learning theory, which emphasizes that deep learning occurs through direct exposure to new environments and reflective observation [8], external training can precisely identify existing deficiencies among medical professionals and address their competency gaps [9]. Moreover, it exposes participants to advanced technologies, complex cases, and direct consultations with leading experts, facilitating the transfer of knowledge from tertiary centers to county-level facilities. Systematic reviews and national program evaluations have confirmed that external training effectively improves physicians’ knowledge, attitudes, and clinical skills [10,11,12]. International evidence from Uganda and Ukraine further supports these findings, with the majority of trainees reporting positive changes in practical concepts and skill implementation upon their return [13,14]. Collectively, these studies validate external training as an effective mechanism for capacity building. However, most of this evidence derives from single-program evaluations, international contexts, or self-organized training initiatives, and it neither specifically targets medical personnel in China’s county hospitals nor examines the effectiveness of external training within the context of national policy. Moreover, few prior studies have employed propensity score matching to control for potential confounding.

The present study therefore aimed to investigate the association between post-policy external training and self-reported competence improvement among county hospital healthcare providers within the context of China’s “Thousand-County Project”. We hypothesized that participants who received external training after policy implementation would report significantly higher competence improvement than those who did not, and that this association would be stronger among providers who were aware of the policy. This hypothesis is grounded in policy feedback theory, which suggests that individuals’ awareness and understanding of a policy can shape their engagement with and response to policy-driven programs [15], and in self-determination theory, which posits that perceived relevance enhances intrinsic motivation and learning outcomes [16]. To test these hypotheses, we analyzed survey data from 605 providers across 30 county hospitals in six provinces, applying propensity score matching, multivariable ordinal logistic regression, interaction tests, and sensitivity analyses.

## 2. Methods

### 2.1. Data and Sampling

#### 2.1.1. Study Design and Sampling

In 2021, China’s National Health Commission (NHC) issued a work plan to improve county hospital capacity and officially established the hospital list. The selection criteria, per the policy document, included five domains: special-region prioritization (e.g., poverty-stricken areas) and service-capacity assessment; clinical specialty capacity; functional positioning in the tiered care system; comprehensive development planning; and local government support. All eligible provinces nominated candidates, and the NHC finalized the list after two review rounds. While the capacity-related criteria may have over-represented better-resourced hospitals, the explicit prioritization of less-developed regions ensured broad geographic coverage. The list thus serves as a national policy implementation framework rather than a random sample, a distinction that should be considered when generalising our findings.

This study employed a multi-center cross-sectional design. The sampling frame comprised 1233 county hospitals included in the national “Thousand-County Project” list. From this framework, we employed a disproportionate stratified sampling strategy, selecting county hospitals from one province within each of the six geographic regions of China: Hebei (North), Heilongjiang (Northeast), Zhejiang (East), Hainan (South Central), Yunnan (Southwest), and Qinghai (Northwest). The number of hospitals sampled from each province was as follows: 4 from Hebei (65 eligible in the province), 6 from Heilongjiang (37 eligible), 7 from Zhejiang (53 eligible), 4 from Hainan (5 eligible), 6 from Yunnan (101 eligible), and 3 from Qinghai (4 eligible). This allocation was based on geographic coverage and survey feasibility, rather than strict proportionality to the number of eligible hospitals in each province.

#### 2.1.2. Participants and Eligibility

All healthcare providers (physicians, nurses, and allied health professionals) working in the selected hospitals during the survey period were eligible to participate. Inclusion criteria were (1) being a licensed healthcare provider; (2) having worked at the current hospital for at least six months; and (3) providing informed consent. Exclusion criteria included: (1) administrative or support staff without direct patient care responsibilities; (2) healthcare providers on extended leave during the survey period; and (3) incomplete questionnaires with missing data on key variables.

#### 2.1.3. Data Collection

All healthcare providers working in the selected hospitals during the survey period were invited to participate. A self-administered structured questionnaire was distributed to healthcare providers in the selected hospitals from June to September 2025. A total of 606 questionnaires were collected. After using the listwise deletion method to remove one case with missing variables, the final analysis sample consisted of 605 participants (with a missing rate < 1%). All participants provided written informed consent prior to participation.

#### 2.1.4. Questionnaire Development

The questionnaire was developed based on a comprehensive literature review of studies on healthcare provider training, competence assessment, and policy implementation in county hospitals. The instrument consisted of four sections: (1) sociodemographic and professional characteristics; (2) training experience; (3) policy awareness; and (4) self-reported competence improvement (measured using a 5-point Likert scale item). We acknowledge that the questionnaire was developed specifically for this study and was not formally validated using psychometric methods such as factor analysis or reliability testing. The competence improvement outcome was measured using a single self-reported item, which does not allow for assessment of internal consistency or test–retest reliability. This is an important limitation, which we have acknowledged in the Discussion.

### 2.2. Study Variables

#### 2.2.1. Exposure Variable

We measured external training status using a single questionnaire item: “After the implementation of the ‘Thousand-County Project’ policy (after January 2022), did you receive external training at a higher-level medical institution?”. Respondents who answered “yes” were classified as the trained group, and those who answered “no” as the untrained group.

Participants self-reported their training status based on their actual experience. We acknowledge that training participation in real-world settings is not randomly assigned and may be influenced by individual-level factors such as professional motivation, clinical performance, or managerial recommendation [17,18,19]. However, detailed information on these selection mechanisms was not available in the survey data, which is a common limitation in cross-sectional observational studies of this nature. To mitigate the potential confounding introduced by non-random training selection, we employed propensity score matching (PSM) to balance observed covariates between the two groups. While PSM cannot eliminate bias from unobserved factors, it substantially reduces observable confounding and improves the comparability of the two groups.

#### 2.2.2. Outcome Variable

The outcome variable of this study is the degree of competence improvement among healthcare providers, assessed through self-report in the questionnaire. The specific item asked: “After the implementation of the ‘Thousand-County Project’ policy (after January 2022), how do you think your competence in disease prevention and management has improved?” Participants responded using a 5-level Likert scale: no improvement, slight improvement, moderate improvement, large improvement, and significant improvement. The assessment covers healthcare providers’ competence in disease prevention, diagnosis, and treatment.

#### 2.2.3. Covariate

Based on the published literature [20,21,22,23,24,25], the following covariates were selected for propensity score matching, as they may influence training opportunities and competence improvement: gender, professional title, major, age (continuous), and years of work experience (continuous). In addition, two other variables were considered potentially associated with the outcomes: awareness of the “Thousand-County Project” policy (not aware vs. aware), and pre-policy external training experience (yes vs. no).

### 2.3. Statistical Analysis

We employed propensity score matching (PSM) to reduce confounding bias by balancing covariates between groups, thereby improving the accuracy of estimating the association [26]. A logistic regression model was constructed with the exposure variable as the dependent variable, including gender, professional title, specialty, age, and work experience as covariates, to estimate the propensity score for each individual. We then performed 1:1 nearest neighbor matching without replacement, setting the caliper at 0.1 SD of the logit propensity score. After evaluating calipers from 0.05 to 0.25, we selected 0.1 as it provided the optimal trade-off between covariate balance and sample size preservation. This caliper value is widely adopted in PSM studies [27,28,29,30].

Descriptive statistics were performed before and after matching. Continuous variables are presented as mean and standard deviation (SD), and categorical variables as counts and percentages.

After successful matching, we fitted a multivariable proportional odds model (ordinal logistic regression) to examine the association between post-policy external training and self-reported competence improvement, using the original 5-point Likert scale as the ordinal outcome. The model simultaneously adjusted for major, professional title, policy knowledge, and pre-policy external training experience, regardless of their statistical significance in univariate analyses, to avoid data-driven variable selection and reduce the risk of omitted-variable bias. Odds ratios (OR) and 95% confidence intervals (CI) were reported. The proportional odds assumption was verified using the Brant test [31].

To assess the robustness of our primary findings, we conducted several sensitivity analyses. First, to account for potential within-hospital correlation, we calculated cluster-robust standard errors at the hospital level [32]. Second, we addressed the potential concern regarding the specification of the PS model. In the primary analysis, we deliberately excluded policy awareness from the propensity score model because we conceptualized it as a potential explanatory factor rather than a pure confounder, and its inclusion might lead to over-adjustment. Given the cross-sectional design, we cannot establish the temporal ordering required for formal mediation analysis; we therefore interpret policy awareness primarily as an effect modifier (as supported by the significant interaction term) rather than a formal mediator. Similarly, we excluded pre-policy external training from the PS model because it was already adjusted for in the post-matching outcome regression. While this post-matching adjustment is not formally equivalent to doubly robust estimation, it helps further reduce residual confounding. To assess the sensitivity of our findings to these decisions, we repeated the PSM with both variables included as additional matching covariates and re-estimated the model. Third, to evaluate the potential influence of unmeasured confounding on our findings, we performed a Rosenbaum bounds sensitivity analysis based on the Wilcoxon signed-rank test for paired ordinal outcomes [33]. This approach retains the full ordinal information of the five-point competence measure and is more appropriate for our outcome than dichotomization. We computed upper-bound *p*-values across a range of Gamma values (from 1.0 to 3.0, in increments of 0.1) to assess the sensitivity of our conclusions to varying degrees of hidden bias.

We further performed interaction analyses to explore whether the association between external training and self-reported competence improvement was modified by policy knowledge or pre-policy external training experience. Interaction terms were entered into separate ordinal logistic regression models, adjusted for the same covariates as the main model, and tested using the Wald test [34].

Data collation was performed using SPSS 27.0, and further statistical analyses were completed with R 4.5.3. *p* < 0.05 was considered statistically significant.

## 3. Results

### 3.1. Propensity Score Matching

Before PSM, a total of 605 healthcare providers were included, with 494 in the untrained group and 111 in the trained group. Subsequently, 1:1 propensity score matching was performed based on gender, professional title, major, age, and years of work experience, resulting in 109 successfully matched pairs. The matching success rate was 98.2%.

In general, SMD < 0.10 indicates good balance after matching. In this study, the SMD of most covariates was less than 0.10, with professional title and gender achieving substantial balance. However, two covariates showed mild imbalance (SMD = 0.188 and 0.110) (Table 1). To address this, we adjusted for these variables in the regression model. A series of subsequent sensitivity analyses yielded consistent results, further supporting the robustness of our findings.

**Figure 1:**
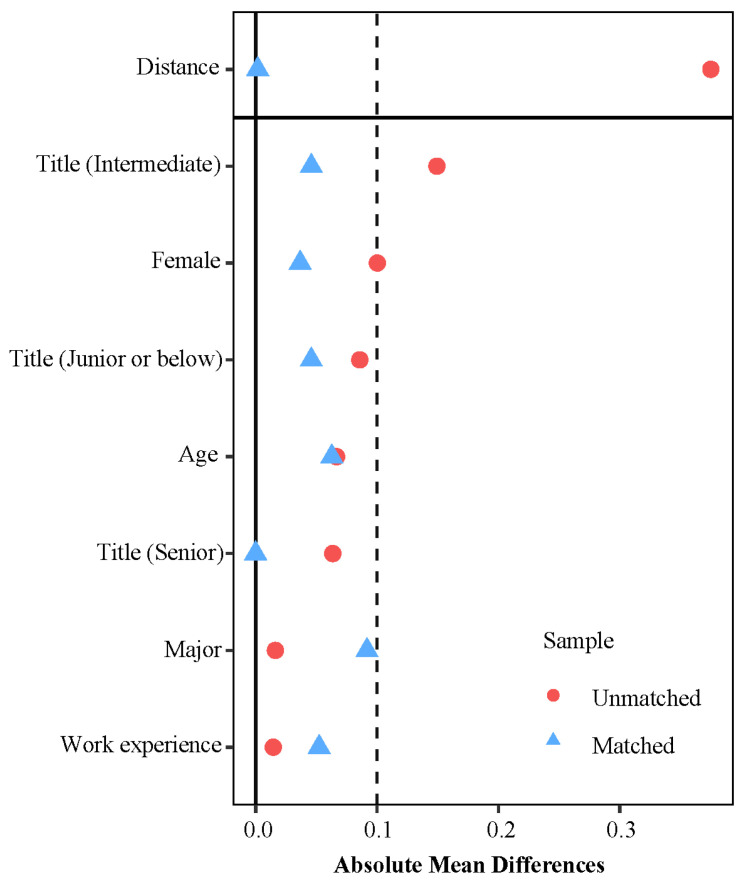
Love plot of absolute mean differences for covariates before and after propensity score matching. The solid vertical line at x = 0.0 marks the reference of perfect balance (zero absolute standardized mean difference); the dashed vertical line at x = 0.1 indicates the predefined balance threshold, below which covariates are typically considered well-balanced.

**Figure 2:**
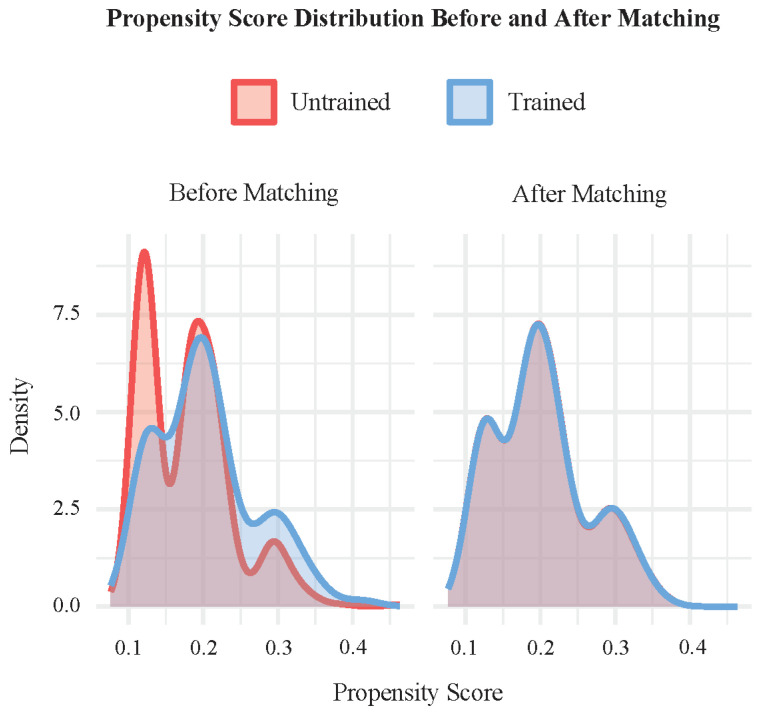
Kernel density plots of propensity scores for the trained and untrained groups before and after matching.

Figure 1 and Figure 2 present the distribution of covariate balance and the estimated propensity score density plots before and after matching, respectively. Before matching, the absolute mean differences for multiple covariates exceeded 0.1, indicating substantial imbalance between the untrained and trained groups. After nearest-neighbor matching, all covariates had absolute mean differences below 0.1. The kernel density plots of the estimated propensity score also showed a high degree of overlap between the two groups, indicating that the matching effectively balanced the covariate distributions between the two groups.

### 3.2. Multivariable Ordinal Logistic Regression Analysis

Table 2 presents the results of the multivariable ordinal logistic regression analysis examining factors associated with self-reported competence improvement after propensity score matching. After adjusting for major, professional title, policy knowledge, and pre-policy external training experience, post-policy external training remained significantly associated with higher competence improvement (OR = 3.36, 95%CI: 1.74–6.48, *p* < 0.001). This association was robust and constituted the primary finding of the analysis. Among the adjusted variables, policy knowledge showed the strongest association with the outcome (OR = 4.50, 95%CI: 2.53–7.99, *p* < 0.001). The Brant test confirmed that the proportional odds assumption was not violated (omnibus χ^2^ = 18.45, df = 18, *p* = 0.43), and the model showed adequate fit (AIC = 570.59).

### 3.3. Sensitivity Analysis

To assess the robustness of our findings, we conducted several sensitivity analyses (Table 3). When accounting for potential within-hospital correlation using cluster-robust standard errors at the hospital level, the association remained virtually unchanged (OR = 3.36, 95%CI: 1.78–6.34, *p* < 0.001). Re-running the propensity score model with policy knowledge and pre-policy external training experience included as additional matching covariates yielded a slightly attenuated but still statistically significant estimate (OR = 2.44, 95%CI: 1.33–4.55, *p* = 0.004). Taken together, these sensitivity analyses consistently supported the primary findings, indicating that the observed association was not sensitive to alternative model specifications or hospital-level clustering.

The Rosenbaum bounds sensitivity analysis was conducted to assess the robustness of the findings to unmeasured confounding (Table 4). The Gamma value represents the minimum strength of association that an unobserved confounder would need to have with the exposure (external training) to completely eliminate the observed association, assuming the confounder is perfectly correlated with the outcome. In our analysis, when Gamma was set to 1.6, the upper-bound *p*-value remained statistically significant (*p* = 0.001), and even at Gamma = 2.4, the upper-bound *p*-value was still below 0.05 (*p* = 0.044). This indicates that an unobserved confounder would need to increase the odds of receiving external training by a factor of 2.4 to fully explain away the observed association, a level of hidden bias that is unlikely given the range of covariates already adjusted for. These results support the robustness of our primary findings to potential unobserved confounding.

### 3.4. Interaction Analyses

To explore whether the association differed across subgroups, we conducted interaction analyses by introducing interaction terms into separate ordinal logistic regression models (Table 5). A significant interaction was observed between post-policy external training and policy knowledge (*p* for interaction = 0.043). Stratified estimates showed that the association was substantially stronger among participants who were aware of the policy (OR = 5.33, 95%CI: 2.18–13.02, *p* < 0.001), whereas no significant association was found among those who were unaware (OR = 1.72, 95%CI: 0.62–4.77, *p* = 0.24). In contrast, the interaction between post-policy external training and pre-policy external training experience was not statistically significant (*p* for interaction = 0.33). Given the modest sample size, these interaction findings should be interpreted with caution.

## 4. Discussion

This study, conducted in the context of China’s “Thousand-County Project” policy, found that participation in external training after the policy was significantly associated with higher self-reported competence improvement among county hospital healthcare providers. Notably, policy awareness showed a strong independent association with competence improvement, and a significant interaction indicated that the association was substantially larger among those who were aware of the policy. These findings were robust to hospital-level clustering and alternative model specifications, supporting the credibility of the observed associations. This study extends previous evidence by highlighting the potential synergy between policy knowledge and external training in the context of China’s county hospital reform.

Healthcare providers who participated in external training after the policy implementation reported significantly higher self-reported competence improvement than those who did not. This finding is consistent with prior studies showing that continuing professional development and external training are associated with improved knowledge and skills among healthcare workers in resource-limited settings [11,12,13,14]. However, most previous studies were conducted within single institutions or specific regions, making it difficult to generalize their findings to a multi-center policy context. Our study extends this evidence by demonstrating a positive association in the specific context of China’s county hospital reform.

Our study also found that policy awareness was associated with higher self-reported competence improvement. An interaction analysis suggested that the association appeared to be stronger among policy-aware providers. This exploratory finding, if confirmed in future studies with larger samples, may indicate a synergistic role of policy knowledge in strengthening the association between external training and competence improvement. Although we did not directly measure motivational constructs in this study, we offer the following possible explanations for the observed interaction, which may inform future research. According to expectancy-value theory, motivation depends on both the expectation of success and the subjective value assigned to a task [35,36]. When healthcare providers understand the policy objectives, they may better perceive the relevance of training to their own competence improvement and to the development of county hospitals, thereby enhancing their subjective task value. An empirical study has shown that policy education has a significant positive impact on medical staff’s policy awareness, which in turn is associated with their ability to adapt to healthcare reforms [37]. Social cognitive theory emphasizes that self-efficacy is a key driver of motivation [38,39]. Providers who are aware of policy goals may be better able to assess the gap between their current competence and the target requirements. Recognizing this gap can stimulate their motivation to improve, and successful training experiences may further enhance their self-efficacy. Moreover, a clear understanding of policy objectives will also motivate them to invest more effort. Studies have clearly indicated that motivation and work engagement among health professionals are critical factors influencing health service delivery, and motivation is closely linked to competency assessment [40]. A cross-sectional study found that medical staff with lower policy alienation are more likely to identify with policies and perceive meaning in their work; those with a strong sense of professional mission tend to be more motivated and goal-oriented [41]. Consequently, healthcare providers who are well-informed about the policy may participate in external training with greater direction and intrinsic motivation, leading to higher learning engagement and greater gains in competence. These explanations are speculative and should be interpreted with caution; future studies that directly measure these psychological constructs are needed to test these hypotheses.

In this study, participants who received external training after policy implementation reported significantly higher competence improvement than those who did not. In contrast, the association between pre-policy external training and competence improvement was not statistically significant. This difference may have multiple explanations. One possibility is that training programs became more relevant or higher in quality after the “Thousand-County Project” was implemented. However, this interpretation remains speculative and warrants further investigation. The National Health Commission explicitly stated in the work plan that county hospitals should accelerate the development of high-quality talent teams and increase talent introduction and training. Subsequently, provinces began implementing external training activities for county hospital healthcare providers. The government may have provided dedicated funding, standardized curricula, and assessment requirements, which may have made training programs more relevant or impactful. Additionally, pre-policy external training may have occurred earlier, and the knowledge and skills acquired might have become outdated or been forgotten. A Danish study found that physicians who continuously participate in medical education are more likely to exhibit intrinsic motivation, such as personal development and professional well-being [42]. Therefore, healthcare providers who had external training before the policy may be more inclined to participate again. They may also possess stronger upward and learning motivation, and some of the observed association may be captured by the post-policy external training variable in the analysis. Both explanations remain speculative and warrant further investigation.

In addition to the quantitative findings, our research team conducted field observations and informal discussions with hospital administrators and healthcare providers during the survey period. These contextual observations, while not systematically collected as part of the formal study data, provide valuable insights into the practical challenges of implementing external training in county hospitals. Some hospitals, benefiting from integrated healthcare alliances or local government programs, were able to send more than 10 healthcare providers annually to high-level hospitals inside or outside the province for 3–6 months of external training, with costs covered by the receiving institutions. In contrast, other hospitals faced severe staffing shortages that prevented healthcare providers from leaving their posts, resulting in very few participants in external training over the past three years. Financial constraints also pose a major challenge. Some hospital managers noted that due to limited funds, training could only be arranged within the province, which they perceived as having limited value for competence improvement. Moreover, even in hospitals that successfully implemented training programs, some managers reported no noticeable improvement in the competence of returnees, citing lack of surgical practice opportunities, inadequate mentoring, and an unsupportive departmental atmosphere as barriers to effective knowledge translation. These practical difficulties suggest that the effectiveness of external training depends not only on the training itself but also on hospital staffing, financial security, and organizational culture. Previous studies have similarly reported that Ugandan doctors trained at Yale University faced financial difficulties and were unable to apply new skills upon return due to resource constraints [13]. Chinese studies have also identified heavy clinical workloads as a barrier to training participation among grassroots healthcare providers [43]. Although our quantitative results indicate that external training is significantly associated with self-reported competence improvement among healthcare providers, some providers may miss training opportunities because of staff shortages and funding limitations. Even among those who receive training, the translation of newly acquired skills into clinical practice may be limited in the absence of a supportive practice environment, follow-up guidance, and organizational support in county hospitals. The contextual observations from our field research highlight that translation of newly acquired skills into clinical practice may be facilitated by supportive practice environments, follow-up guidance, and organizational support. These implications should be interpreted as hypothesis-generating rather than definitive policy recommendations, given the observational nature of our study.

The study sample has good geographic representativeness and can offer real-world evidence for examining the association between external training and self-reported competence improvement in this policy context. Moreover, the application of propensity score matching helps reduce observable confounding, yielding more reliable estimates than conventional regression approaches. However, several limitations should be acknowledged. First, competence improvement was measured using a single self-reported item, rather than a validated multi-dimensional scale. Although we employed anonymous questionnaires and clear confidentiality commitments to reduce social desirability bias, self-reported measures may still be subject to recall bias and subjective assessment. Notably, social desirability bias could lead trained participants to overreport improvement, potentially inflating the observed association; however, such biases are generally non-differential and would tend to attenuate rather than exaggerate the true association. Moreover, the single-item measure does not allow for assessment of internal consistency or reliability, as the questionnaire was developed specifically for this study and was not formally validated using psychometric methods. Future research should consider incorporating objective competence indicators or validated instruments. Second, although propensity score matching balanced most covariates, residual imbalance persisted for a few variables. We adjusted for these variables in the subsequent regression model to minimize their impact. Nevertheless, residual confounding from imperfectly balanced covariates cannot be entirely excluded, and the results should be interpreted with this in mind. Additionally, as with all observational studies, the possibility of unmeasured confounding from unobserved factors cannot be completely ruled out. To assess this concern, we conducted a Rosenbaum bounds sensitivity analysis, which suggested that our findings were reasonably robust to unobserved confounding. The cross-sectional nature of our study also precludes causal inference. Future studies employing longitudinal designs could further strengthen causal inference by tracking competence changes before and after training. Third, the matching procedure reduced the effective sample size, which may limit statistical power for detecting small effects or interaction terms. However, the main association remained statistically significant, and the distribution of key covariates was well balanced after matching, suggesting that the sample loss did not introduce serious selection bias. Forth, the exposure variable was measured as a binary indicator of whether participants had received external training, without collecting detailed information on training duration, content, host institution, specialty relevance, or mentoring intensity. Future studies should collect such information to enable dose–response analyses and to identify which specific types of external training are most effective in improving healthcare providers’ competence.

## 5. Conclusions

This multi-center cross-sectional study examined the association between post-policy external training and self-reported competence improvement among county hospital healthcare providers in the context of China’s “Thousand-County Project,” using propensity score matching and ordinal logistic regression to reduce confounding and preserve the ordinal outcome. External training was significantly associated with higher self-reported competence improvement, and this association was substantially stronger among those who were aware of the policy. These findings were robust to alternative model specifications and sensitivity analyses. Given the cross-sectional design and the use of a single self-reported item to measure competence improvement, these findings should be interpreted as associations rather than causal effects. Although we employed several analytical approaches to minimize bias, the possibility of residual confounding cannot be entirely excluded.

These findings have several potential implications. The observed association suggests that continued support for external training may be a valuable strategy for enhancing healthcare providers’ competence in county hospitals. In addition, the significant interaction with policy awareness implies that strengthening policy dissemination, through seminars, training sessions, or other communication channels, could potentially reinforce the benefits of external training. However, these implications are hypothesis-generating and should be interpreted with caution, as the observational design does not support strong causal claims. Future research employing longitudinal designs, objective measures of professional competence, and validated assessment instruments is needed to establish more robust temporal relationships between external training and competence improvement and to provide stronger evidence for informing policy decisions aimed at strengthening county hospital capacity.

## Figures and Tables

**Table 1:** Characteristics before and after propensity score matching.

	Before Propensity Score Matching (*n* = 605)			After Propensity Score Matching (*n* = 218)		
Variables	Untrained (*n* = 494)	Trained (*n* = 111)	SMD	Untrained (*n* = 109)	Trained (*n* = 109)	SMD
Professional title (%)			0.307			0.110
Junior or below	167 (33.8%)	28 (25.2%)		33 (30.3%)	28 (25.7%)	
Intermediate	171 (34.6%)	55 (49.5%)		48 (44.0%)	53 (48.6%)	
Senior	156 (31.6%)	28 (25.2%)		28 (25.7%)	28 (25.7%)	
Years of work experience (mean (SD))	14.06 (10.02)	13.96 (7.19)	0.012	13.72 (8.39)	14.10 (7.18)	0.048
Major (%)			0.034			0.188
Clinical Medicine	170 (34.4%)	40 (36.0%)		49 (45.0%)	39 (35.8%)	
Other	324 (65.6%)	71 (64.0%)		60 (55.0%)	70 (64.2%)	
Age (mean (SD))	38.26 (8.74)	38.77 (7.60)	0.062	38.15 (8.06)	38.62 (7.53)	0.061
Gender (%)			0.206			0.074
Male	173 (35.0%)	50 (45.0%)		52 (47.7%)	48 (44.0%)	
Female	321 (65.0%)	61 (55.0%)		57 (52.3%)	61 (56.0%)	

Exposure variable: whether the individual participated in external training after the policy implementation.

**Table 2:** Multivariable ordinal logistic regression analysis of factors associated with competence improvement after propensity score matching.

Variable	OR (95%CI)	*p*-Value
Major		
Clinical Medicine (ref: other)	0.99 (0.58, 1.70)	0.976
Professional title		
Intermediate (ref: Junior or below)	0.63 (0.34, 1.17)	0.144
Senior (ref: Junior or below)	1.07 (0.50, 2.27)	0.869
Policy knowledge		
Aware (ref: unaware)	4.50 (2.53, 7.99)	<0.001
Pre-policy external training		
Yes (ref: no)	1.25 (0.64, 2.43)	0.511
Post-policy external training		
Yes (ref: no)	3.36 (1.74, 6.48)	<0.001

**Table 3:** Sensitivity analyses for the association between post-policy external training and competence improvement.

Model	OR (95%CI)	*p*-Value
Main Model	3.36 (1.74, 6.48)	<0.001
Robust Standard Error for Hospital Clustering	3.36 (1.78, 6.34)	<0.001
New PSM (incorporating Policy knowledge and Pre-policy external training)	2.44 (1.33, 4.55)	0.004

**Table 4:** Rosenbaum bounds sensitivity analysis.

Gamma	sig+ (Upper Bound)	sig− (Lower Bound)
1.0	<0.001	<0.001
1.1	<0.001	<0.001
1.2	<0.001	<0.001
1.3	<0.001	<0.001
1.4	<0.001	<0.001
1.5	<0.001	<0.001
1.6	0.001	<0.001
1.7	0.002	<0.001
1.8	0.004	<0.001
1.9	0.006	<0.001
2.0	0.010	<0.001
2.1	0.016	<0.001
2.2	0.023	<0.001
2.3	0.032	<0.001
2.4	0.044	<0.001
2.5	0.059	<0.001
2.6	0.075	<0.001
2.7	0.095	<0.001
2.8	0.117	<0.001
2.9	0.141	<0.001
3.0	0.167	<0.001

**Table 5:** Interaction analyses between post-policy external training and key covariates.

Subgroup	OR (95%CI)	*p*-Value
Post-policy external training × Policy knowledge		
Not aware	1.72 (0.62, 4.77)	0.24
Aware	5.33 (2.18, 13.02)	<0.001
*p*-value for the interaction term		0.043
Post-policy external training × Pre-policy external training		
No	2.51 (1.05, 6.02)	—
Yes	4.76 (1.94, 11.68)	—
*p*-value for the interaction term		0.33

Since the interaction term was not significant (*p* = 0.33), the differences in stratified ORs between groups were not statistically significant; therefore, the stratified *p*-values are not reported separately.

## Data Availability

The data presented in this study are available on request from the corresponding author due to privacy and ethical restrictions.
